# Epidemiological characteristics of four common respiratory viral infections in children

**DOI:** 10.1186/s12985-020-01475-y

**Published:** 2021-01-06

**Authors:** Guohong Zhu, Dan Xu, Yuanyuan Zhang, Tianlin Wang, Lingyan Zhang, Weizhong Gu, Meiping Shen

**Affiliations:** 1 Department of Pulmonology, The Children’s Hospital, Zhejiang University School of Medicine, National Clinical Research Center for Child Health, 3333, Binsheng Road, Hangzhou, Zhejiang Province China; 2 Department of Internal Medicine, The Children’s Hospital, Zhejiang University School of Medicine, National Clinical Research Center for Child Health, Hangzhou, Zhejiang Province China; 3 Department of Pathology, The Children’s Hospital, Zhejiang University School of Medicine, National Clinical Research Center for Child Health, Hangzhou, Zhejiang Province China; 4 Nursing Department, The Children’s Hospital, Zhejiang University School of Medicine, National Clinical Research Center for Child Health, Hangzhou, Zhejiang Province China

**Keywords:** Respiratory infection, Children, Respiratory virus, Adenovirus, Influenza, Respiratory syncytial virus

## Abstract

**Background:**

Viruses are the main infectious agents of acute respiratory infections in children. We aim to describe the epidemiological characteristics of viral pathogens of acute respiratory tract infections in outpatient children.

**Methods:**

From April 2018 to March 2019, the results of viral detection using oral pharyngeal swabs from 103,210 children with acute respiratory tract infection in the outpatient department of the Children’s Hospital, Zhejiang University School of Medicine, were retrospectively analyzed. Viral antigens, including adenovirus (ADV), influenza A (FLUA), influenza B (FLUB) and respiratory syncytial virus (RSV), were detected by the colloidal gold method.

**Results:**

At least one virus was detected in 38,355 cases; the positivity rate was 37.2%. A total of 1910 cases of mixed infection with two or more viruses were detected, and the positivity rate of multiple infection was 1.9%. The ADV positivity rate was highest in the 3–6-year-old group (18.7%), the FLUA positivity rate was highest in the > 6-year-old group (21.6%), the FLUB positivity rate was highest in the > 6-year-old group (6.6%), and the RSV positivity rate was highest in the < 1-year-old group (10.6%). There was a significant difference in the positivity rate of viral infection among different age groups (χ^2^ = 1280.7, *P* < 0.001). The rate of positive viral infection was highest in winter (47.1%). The ADV infection rate was highest in spring (18.2%). The rates of FLUA and FLUB positivity were highest in winter (28.8% and 3.6%, respectively). The rate of RSV positivity was highest in autumn (17.4%). The rate of positive viral infection in different seasons was significantly different (χ^2^ = 6459.1, *P* < 0.001).

**Conclusions:**

Viral infection rates in children differ for different ages and seasons. The positivity rate of ADV is highest in the preschool period and that of RSV is highest in infants; that of FLU increases with age. The total positive rate of viral infection in different seasons is highest in winter, as is the rate of FLU positivity.

## Background

Acute respiratory infections (ARIs) are common respiratory diseases in childhood. ARIs are the main cause of morbidity, hospitalization and death in children and cause economic losses to both families and society [1–3]. Viruses are the main infectious agents of ARIs [4], among which respiratory syncytial virus (RSV), rhinovirus, human metapneumovirus, parainfluenza virus, human enterovirus, influenza virus (FLU), human coronavirus, adenovirus (ADV) and human Boca virus account for approximately 70% of ARI viral infections [2]. Studies have analyzed viral infections in ARIs. A systematic review and meta-analysis examined some viral infections in children under 5 years of age hospitalized for acute lower respiratory infections (ALRIs) and found that influenza viruses (IVs), parainfluenza viruses (PIVs), ADVs and coronaviruses (CVs) were all commonly responsible for ALRIs [5]. RSV is also a common cause of childhood ALRI and a major cause of hospital admissions in young children [6].

Our hospital is the only third-class children's hospital in Zhejiang Province. The daily outpatient volume is more than 6000, and children from all over the province visit the facility. To understand the epidemic characteristics of ARIs in Zhejiang Province, we analyzed ADV, FLUA, FLUB and RSV detection rates in outpatients from April 2018 to March 2019.

## Methods

From April 1st, 2018, to March 31st, 2019, 103,210 children were diagnosed with ARIs in the outpatient clinic at the Children’s Hospital, Zhejiang University School of Medicine.

The hospital is in Hangzhou, the capital of Zhejiang. Zhejiang Province, located on the southeast coast of China, has a typical subtropical monsoon climate with four distinctive seasons.

1. *Sample collection* Oropharynx swabs were collected by a nurse (0.5 ml sample extraction buffer was added to the sampling tube in advance) and immediately sent to the Pathology Department of our hospital for detection.
2. *Detection of viral antigens* The viral antigens ADV, FLUA, FLUB and RSV were detected by the colloidal gold method (Kaibili respiratory virus antigen detection kit, Hangzhou Genesis Corporation). The results could be observed within 15 min.
3. *Statistical analysis* The chi-square test was used to compare the classified variable groups. *P* < 0.05 was considered statistically significant. Data were analyzed by SAS 9.4.

## Results

1. Overall viral detection
  1. Among 103,210 cases, 38,355 were positive for at least one virus, with a total positivity rate of 37.2%. There were 56,250 males and 46,960 females, with an average age of 3.5 ± 2.6 years; 13,984 were under 1 year old, 39,552 were 1–3 years old, 32,731 were 3–6 years old, and 16,943 were over 6 years old. Positive cases involved 21,374 (38.0%) males and 16,981 (36.2%) females, and the positivity rate differed significantly between the sexes (χ^2^ = 37.0, *P* < 0.001).
  2. Proportion of viral infection
    Among the 38,355 virus-positive children, the FLUA infection rate was highest (18.2%), followed by ADV (13.0%), RSV (7.0%), and FLUB (2.3%) (see Table 1 for details). Patients infected by two or more viruses (mixed infection) are also listed in Table 1.

**Table 1:** Viral infection in acute respiratory infections in children

Viruses infected	n
ADV	13,375 (13.0%)
FLUA	17,509 (18.2%)
FLUB	2187 (2.3%)
RSV	7223 (7.0%)
ADV and FLUA	926 (0.9%)
ADV and FLUB	83 (0.1%)
ADV and RSV	721(0.7%)
FLUA and FLUB	3
FLUA and RSV	69 (0.1%)
FLUB and RSV	2
Three kinds	21
Four kinds	4

*ADV* adenovirus, *FLUA* influenza A, *FLUB* influenza B, *RSV* respiratory syncytial virus
  3. Viral infection at different ages
    Among the 38,355 children with viral infection positivity, the difference among different age groups was statistically significant (χ^2^ = 1280.7, *P* < 0.001, see Table 2 for details).

**Table 2:** The positivity rate of four viruses in different age groups

Virus age	(n, %)	χ^2^	*P* value
ADV	13,375 (13.0)	2601.2	< 0.001
< 1 year	523 (3.7)		
1–3 years	3862 (9.8)		
3–6 years	6107 (18.7)		
> 6 years	2883 (17.0)		
FLUA	17,509 (18.2)	535.3	< 0.001
< 1 year	1658 (12.8)		
1–3 years	6229 (16.8)		
3–6 years	6135 (20.4)		
> 6 years	3487 (21.6)		
FLUB	2187 (2.3)	1869.3	< 0.001
< 1 year	79 (0.6)		
1–3 years	329 (0.9)		
3–6 years	707 (2.4)		
> 6 years	1072 (6.6)		
RSV	7223 (7.0)	2046.6	< 0.001
< 1 year	1481 (10.6)		
1–3 years	4016 (10.2)		
3–6 years	1534 (4.7)		
> 6 years	192 (1.1)		
Total		1280.7	< 0.001
< 1 year	3684 (26.3)		
1–3 years	13,834 (35.0)		
3–6 years	13,554 (41.4)		
> 6 years	7283 (43.0)		

**Fig. 1:**
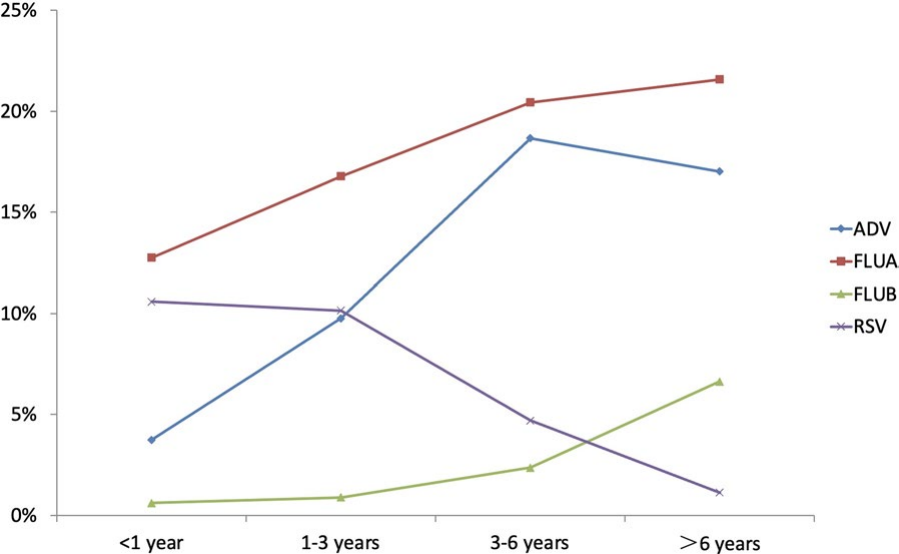
Positivity rates of four viruses in different age groups
  2. Positivity rate in different seasons
    The positivity rates of the four viruses in spring, summer, autumn and winter were 18.2%, 16.0%, 12.6% and 11.2% for ADV, 0.4%, 0.2%, 2.7% and 28.8% for FLUA, 0.2%, 0.1%, 0.0% and 3.6% for FLUB, and 3.5%, 3.4%, 17.4% and 5.6% for RSV, respectively. Figure 2 shows that the total detection rate was highest in winter; ADV was highest in spring, FLUA and FLUB were highest in winter, and RSV was highest in autumn.

*ADV* adenovirus, *FLUA* influenza A, *FLUB* influenza B, *RSV* respiratory syncytial virus
  4. Viral infection in different seasons
    Positivity rates in spring, summer, autumn and winter were 19.8%, 18.7%, 30.2% and 47.1%, respectively, with the highest rates in winter.
2. Detection of each virus
  1. Positivity rate of different age groups
    The positivity rates of the four viruses in different age groups are shown in Table 2. Figure 1 shows that the rate of ADV positivity was highest in the 3–6-year-old group; the rates of FLUA and FLUB increased with increasing age, and that of RSV was highest in the < 1-year-old group.

**Fig. 2:**
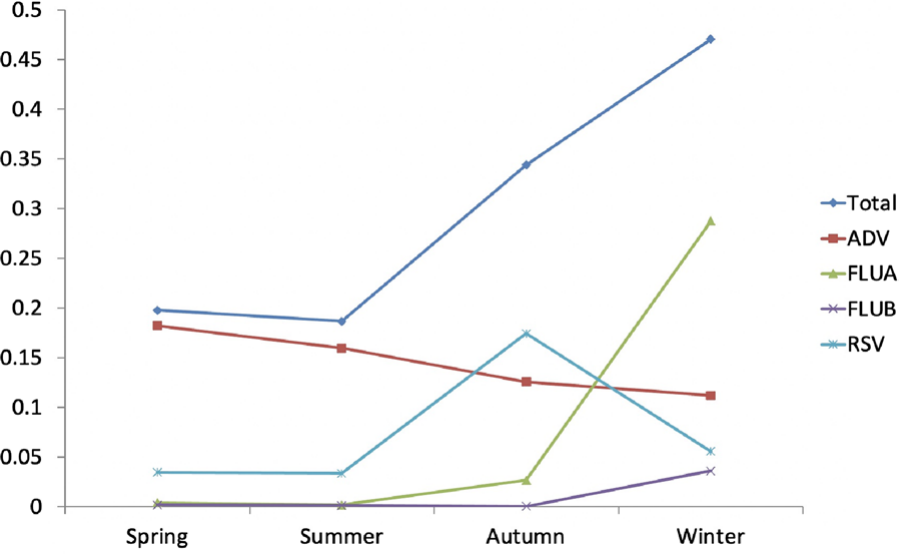
Positivity rates of four viruses in different seasons
  3. Monthly positive detection of each virus
    The positivity rates of the four viruses among the 38,355 patients are shown in Fig. 3. The highest positivity rate of ADV occurred in May, FLUA in February, FLUB in March and RSV in December. At the same time, the overall positivity rate was highest in February.

**Fig. 3:**
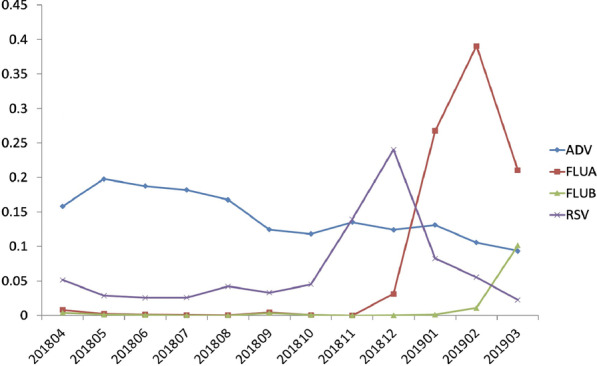
The positivity rate of four viruses in each month

## Discussion

Acute respiratory infections (ARIs) are a major health problem worldwide. Respiratory viruses are the main cause of infection [2]. Nasopharynx swabs or oropharynx swabs are commonly used for pathogen detection of ARIs [3, 7, 8]. In this study, the detection of four respiratory viruses (ADV, FLUA, FLUB, and RSV) using oropharyngeal swabs from 103,210 children with ARIs was analyzed retrospectively. Among them, 38,355 cases were positive for at least one virus, with a total positivity rate of 37.2%, which is close to the rates of respiratory viruses in the literature (32.5–35.8%) [7, 9]. Other studies have reported higher (69.1–85.8%) and lower (14.6–24.5%) rates [4, 10–12]. There were 1910 cases infected with two or more viruses, and the positivity rate of multiple infection was 1.9%, close to the 2.2% reported by Krishnan [13] but lower than the 5.0% reported by Kim [14]. Different analyses of positivity rates are related to different kinds of patients, geographical areas and detection methods. Among the 38,355 patients, 38.0% were male and 36.2% female. The difference between the two sexes was statistically significant (χ^2^ = 37.0, *P* < 0.001, see Table 1). The literature [3, 9] reports no significant differences between males and females in common respiratory viral infection, though Krishnan A and Shapiro d [13, 15] found that the detection rate of males was higher than that of females, which was consistent with our results.

The rates of ADV, FLUA, FLUB and RSV positivity were 13.0%, 18.2%, 2.3% and 7.0%, respectively. The highest rate of FLUA positivity indicated a prevalence of FLUA in Zhejiang Province from April 2018 to March 2019. In our study, the total positivity rate was the highest in winter, while the peak of viral detection was in spring, as reported by Wang [12]; no significant difference between seasons was reported by Kurskaya [3]. The difference my due to local geography and climate. The rate of positive viral infection in different age groups increased with age, with a statistically significant difference. Wang [12] reported that 92.8% of children positive for viral pathogens were ≤ 3 years old. Kim [14] reported that respiratory viruses mainly occurred in infants and children under 5 years old. The types of viruses analyzed and different detection methods may be related to different age distributions.

The rate of ADV positivity was highest in the 3–6-year-old group (18.7%) and in May (19.8%). Kurskaya [3] reported no significant difference in the distribution of ADV among different age groups. Kim [14] found that the detection rate of ADV was highest in the 1–5-year-old group but Chen [16] that patients aged 1–6 had the highest rate of ADV positivity. Calvo [17] reported a prevalence of ADV infection in November and December, though according to Kim [14], ADV did not show significant seasonality. Botti [18] indicated that ADV is mainly prevalent in summer.

The rates of FLUA and FLUB positivity increased with increasing age. Both the highest rates of FLUA and FLUB were in the 6-year-old group (21.6% and 6.6%, respectively). The highest rates of FLUA and FLUB positivity occurred in February and March, respectively, both in winter. Kurskaya and Machablishvili [3, 8] reported that the rate of influenza positivity increased with age, which is consistent with our results. Kurskaya and Dong [3, 11] also found that the highest detection rate of influenza occurred in winter, and Althouse [19] reported that the peak of influenza was in the middle of spring (April to June). Our results show that FLUA was the dominant factor in influenza, yet Luniewska [20] reported that FLUA and FLUB were both dominant in the epidemic season.

The RSV positivity rate was highest in the group less than 1 year old (10.6%) and highest in December (24.1%). Kurskaya and Richter [3, 10] also reported that the RSV positivity rate decreased with increasing age. Choi [4] found that children under 1 year old mainly accounted for RSV positivity, which was consistent with our results. Choi [4] also reported that the rate of RSV positivity peaked in autumn and winter, while Althouse [19] reported that the peak was in late summer and early autumn. The similarities and differences in rates for each virus may be related to climate and environmental factors, population distribution, economic status, diagnostic methods used, and accessibility and connectivity of medical care [3, 21]. High population density and an increase in shared space are conducive to the spread of influenza and other viruses. Therefore, it is a general recommendation to avoid going to school or work during infection [9]. January to February 2019 occurred during the end of the semester in primary and secondary schools in Zhejiang Province. Although there was an increase in patients with fever, schools were not closed. This may be one of the reasons for the high rate of influenza positivity during this period.

In clinical practice, it is difficult to distinguish bacterial from viral infection in ARIs in children. Doctors are concerned about bacterial infection, especially in infants. This could lead to the use of unnecessary antibiotics [4, 22]. We used the colloidal gold method to detect virus antigens of ADV, FLUA, FLUB and RSV. The results could be observed within 15 min, providing clinicians with timely antiviral treatment to avoid unnecessary antibiotic prescription.

*Limitations* (1) As our study is a retrospective study and not a prospective analysis, there are the following deficiencies. (1) Due to objective reasons, we did not carry out influenza testing from October 12th to December 2nd, 2018. Although influenza surveillance during the same period revealed only sporadic cases, a continuous test would be better. (2) Our report showed that the ADV positivity rate was highest in the 3–6-year-old group and that the rate of influenza positivity increased with age. Calvo [17] reported that children with ADV infection who need to be hospitalized are usually under 2 years old. Chen [16] reported that severe and lethal cases of ADV were under 2 years old. Zhang [23] found that most children with severe influenza were younger than 5 years old. In addition, the literature [24] suggests that male sex and under 6 months are risk factors related to RSV hospitalization. This study did not analyze the corresponding clinical characteristics of the children. (3) Rhinovirus, parainfluenza virus and other common viruses causing ARIs in children were not detected. (2) Seasonal variation of influenza infection is very large. The prevalence of viruses causing ARIs varies with time in different areas and years [3, 20]. Therefore, our 1-year detection research cannot reflect the seasonal pattern of respiratory virus in other years.

## Conclusion

In conclusion, our single-center study showed that among ARIs, the rate of ADV positivity was highest in preschool children. RSV also had the highest positivity rate among infants. The rate of influenza positivity increased with age. The total positivity rate of the four viral infections was highest in winter.

## Data Availability

Not applicable.

## Abbreviations

ARIs: Acute respiratory infections
ADV: Adenovirus
FLUA: Influenza A
FLUB: Influenza B
RSV: Respiratory syncytial virus
